# Genome sequence data of *Bacillus* sp. CCB-MMP212 isolated from Malaysian mangrove: A potential strain in arsenic resistance with ArsI, C•As lyase

**DOI:** 10.1016/j.dib.2022.108597

**Published:** 2022-09-13

**Authors:** Nor Azura Azami, Nyok-Sean Lau, Go Furusawa

**Affiliations:** Centre for Chemical Biology, Universiti Sains Malaysia, Bayan Lepas, Penang, Malaysia

**Keywords:** Bacillus, Genome sequence, Arsenic resistance, Mangrove

## Abstract

*Bacillus* sp. CCB-MMP212 is a Gram-positive bacterium isolated from mangrove sediment in Matang Perak, Malaysia (4.85496°E, 100.73495°N). Genome sequencing was performed using the Oxford Nanopore and Illumina platforms. The assembled genome was annotated using the rapid annotation subsystem technology server (RAST) (rast.nmpdr.org). The genome size of the *Bacillus* sp. CCB-MMP212 was 6,151,644 base pairs (bp) with a G+C content of 34.75%. The genome includes 6,311 coding sequences and 58 RNAs. The sequence has been deposited at Genbank with the accession number of JALDQE000000000. Interestingly, an arsenic resistance (ars) operon consisted of arsenic resistance operon repressor (*ars*R), ACR3 family arsenite efflux transporter (*ars*B), and arsenate reductase (*ars*C) genes were found in the genome. In addition, the arsenic inducible gene (*ars*I), which encoded a dioxygenase with C•As lyase activity, was also found in the ars operon. The enzyme is crucial for the methylation of methylarsonous acid [MAs(III)] and trivalent roxarsone [Rox(III)]. This dataset reveals the genetic ability of this strain in arsenic resistance. To the best of our knowledge, the *ars*I encoding C•As lyase is rarely reported within the genus *Bacillus*. Therefore, the dataset presented in this manuscript provides further insight into the arsenic resistance mechanisms of the genus *Bacillus*.

**Specification Table**

**Table:** 

Subject	Biology
Specific subject area	Microbiology, Genomics and Molecular Biology
Type of data	Tables, Figures and whole-genome sequencing data
How data were acquired	The complete genome sequence was determined using the Oxford Nanopore and Illumina platforms
Data format	Raw and analysed
Parameters for data collection	Pure culture of *Bacillus* sp. CCB-MMP212 was grown in marine agar (MA) at a temperature of 30°C and a pH of 7
Description of data collection	The genomic DNA was sequenced using the Oxford Nanopore and Illumina platforms, while subsequence annotation was done using the RAST server (RAST)
Data source location	Sediment samples were collected from Matang mangrove forest, Perak, Malaysia
Data accessibility	The complete genome sequence of *Bacillus* sp. CCB-MMP212 was deposited in NCBI GenBank under accession number JALDQE000000000 Direct URL to data: https://www.ncbi.nlm.nih.gov/nuccore/JALDQE000000000 Database link: BioProject: PRJNA818481 BioSample: SAMN26865143

## Value of the Data

• The whole-genome sequence of *Bacillus* sp. CCB-MMP212 could provide valuable information to researchers working on the *Bacillus* strain with the potential for arsenic resistance.
• The *Bacillus* sp. CCB-MMP212 could be a referral strain for the *ars*I encoding C•As lyase in the genus *Bacillus*.
• The whole-genome sequence of *Bacillus* sp. CCB-MMP212 can contribute to the understanding of molecular information and related characteristics of this strain.
• The data can be used by researchers working in the field of Microbiology, Genomics, and Molecular Biology.

## 1 Data Description

**Fig. 1:**
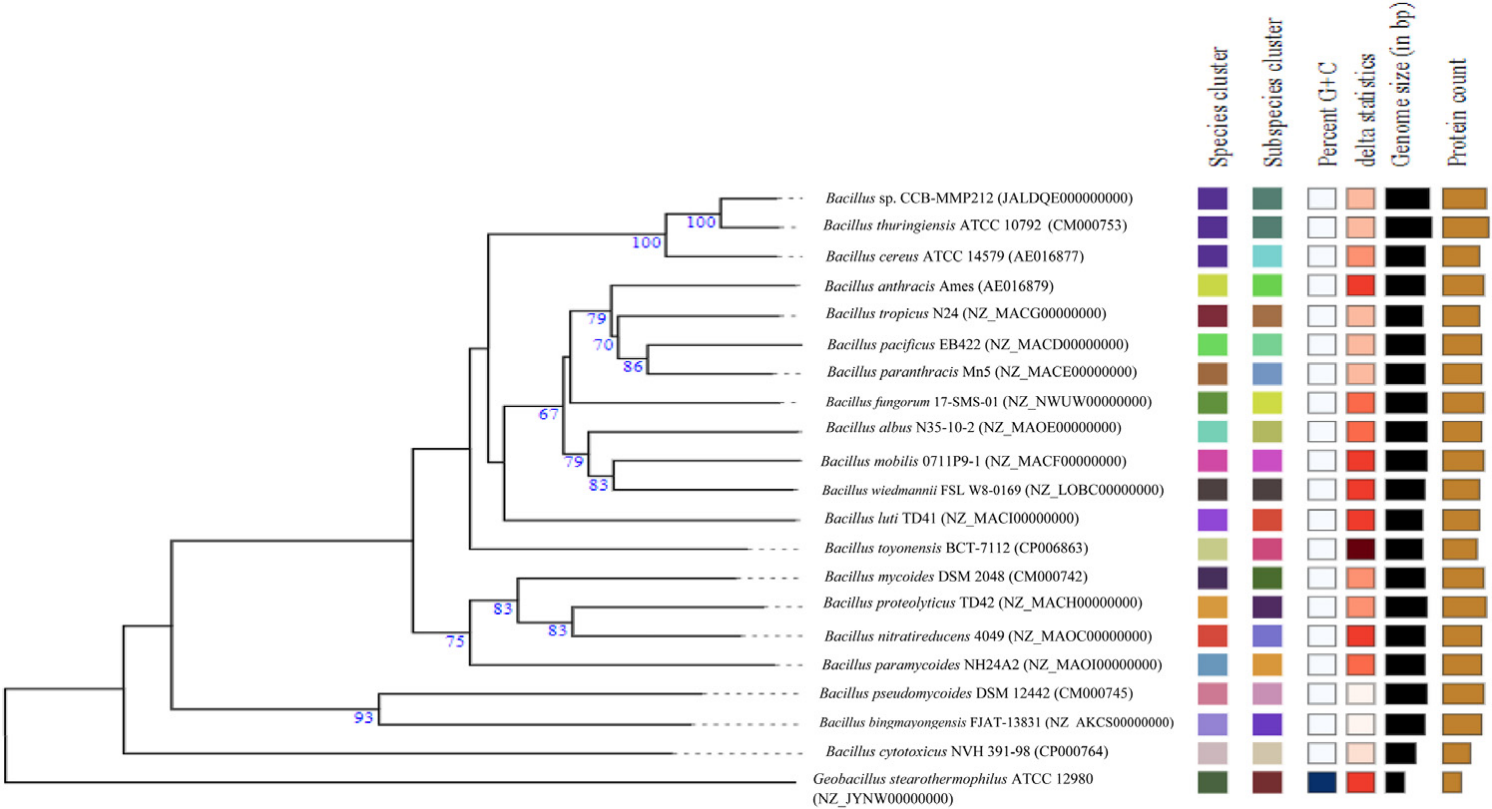
Whole genome phylogenetic tree constructed by Type Strain Genome Server, using Maximum Likelihood Method based on Generalised Time Reversible (GTR) model. The tree shows the close relationship between *Bacillus* sp. CCB-MMP212 with the closed species, while *Geobacillus stearothermophilus* ATCC 12980 is included to serve as an outgroup.

*Bacillus* sp. CCB-MMP212 was isolated from mangrove sediment during the microbial diversity investigation of Matang Mangrove Forest, Perak, Malaysia. This study presents the complete whole-genome sequence of *Bacillus* sp. CCB-MMP212. The genome sequencing was performed using the Oxford Nanopore and Illumina platforms. The assembled genome was annotated using the rapid annotation with the RAST server (RAST) (rast.nmpdr.org) [1]. The result shows that the genome contained 6,151,644 base pairs (bp) with a G+C content of 34.75%. The genome includes 6,311 coding sequences and 58 RNAs. The assembly statistics and genomic features of *Bacillus* sp. CCB-MMP212 were summarised in Table 1. *Bacillus* sp. CCB-MMP212 whole-genome sequence was used to construct an accurate evolutionary relationship with other bacterial whole genomes closely related to *Bacillus* species using the Type Strain Genome Server, (TYGS) (https://tygs.dsmz.de) [2]. Fig. 1 shows that *Bacillus* sp. CCB-MMP212 is closely related to *Bacillus thuringiensis* ATCC 10792 and forms a clade with *Bacillus cereus* ATCC 14579. To confirm the phylogenetic relationship of CCB-MMP212, the average nucleotide identity (ANI) values and Digital DNA-DNA hybridization (dDDH) values between *Bacillus* sp. CCB-MMP212 and closely related species were calculated by the OrthoANI algorithm [3] and TYGS, respectively. Table 2 shows the ANI value of *Bacillus* sp. CCB-MMP212 and *Bacillus thuringiensis* ATCC 10792 exhibited the highest percentage (98.19%), followed by *Bacillus cereus* ATCC 14579 with an ANI value of 96.66%. From the Table, the ANI values of other strains were below the species boundary value (ANI, >95%) [4]. The dDDH values of *B. thuringiensis* ATCC 10792 (84.4%) and *B. cereus* ATCC 14579 (71.5%) were higher than the species boundary value (<70%) (Table 2) [5], indicating the consistency of the phylogenetic relationship of *Bacillus* sp. CCB-MMP212.

**Table 1:** Assembly statistics and genomic features of *Bacillus* sp. CCB-MMP212.

Contigs no.	41
Genome size (bp)	6,151,644
GC content (%)	34.75
Largest contig (bp)	933547
N50 contig (bp)	556935
N75 contig (bp)	211718
L50 contig	5
L75	10
Number of Coding Sequences	6311
Number of RNAs	58
Number of subsystems	353
NCBI Accession No	JALDQE000000000

**Table 2:** Comparison of several *Bacillus* isolates based on genomic metrics including digital DNA-DNA hybridization (dDDH) and average nucleotide identity (ANI).

	dDDH (d4, in %)	ANI (%)	NCBI accession
*Bacillus* *thuringiensis* ATCC 10792	84.4	98.19	CM000753
*Bacillus* *cereus* ATCC 14579	71.5	96.66	AE016877
*Bacillus toyonensis* BCT-7112	45.0	91.61	CP006863
*Bacillus* *tropicus* N24	44.9	91.78	NZ_MACG00000000
*Bacillus* *fungorum* 17-SMS-01	44.5	91.52	NZ_NWUW00000000
*Bacillus* *paranthracis* Mn5	44.3	91.48	NZ_MACE00000000
*Bacillus* *wiedmannii* FSL W8-0169	44.0	91.29	NZ_LOBC00000000
*Bacillus* *anthracis* Ames	43.8	91.41	AE016879
*Bacillus* *luti* TD41	43.5	91.30	NZ_MACI00000000
*Bacillus* *pacificus* EB422	43.4	91.28	NZ_MACD00000000
*Bacillus* *albus* N35-10-2	43.1	91.13	NZ_MAOE00000000
*Bacillus* *mobilis* 0711P9-1	42.6	91.01	NZ_MACF00000000
*Bacillus* *proteolyticus* TD42	39.5	89.85	NZ_MACH00000000
*Bacillus* *nitratireducens* 4049	39.3	89.77	NZ_MAOC00000000
*Bacillus* *mycoides* DSM 2048	38.3	89.43	CM000742
*Bacillus* *paramycoides* NH24A2	37.1	88.97	NZ_MAOI00000000
*Bacillus* *pseudomycoides* DSM 12442	26.9	82.28	CM000745
*Bacillus* *bingmayongensis* FJAT-13831T	26.8	82.53	NZ_AKCS00000000
*Bacillus* *cytotoxicus* NVH 391-98	25.5	81.38	CP000764

**Fig. 2:**
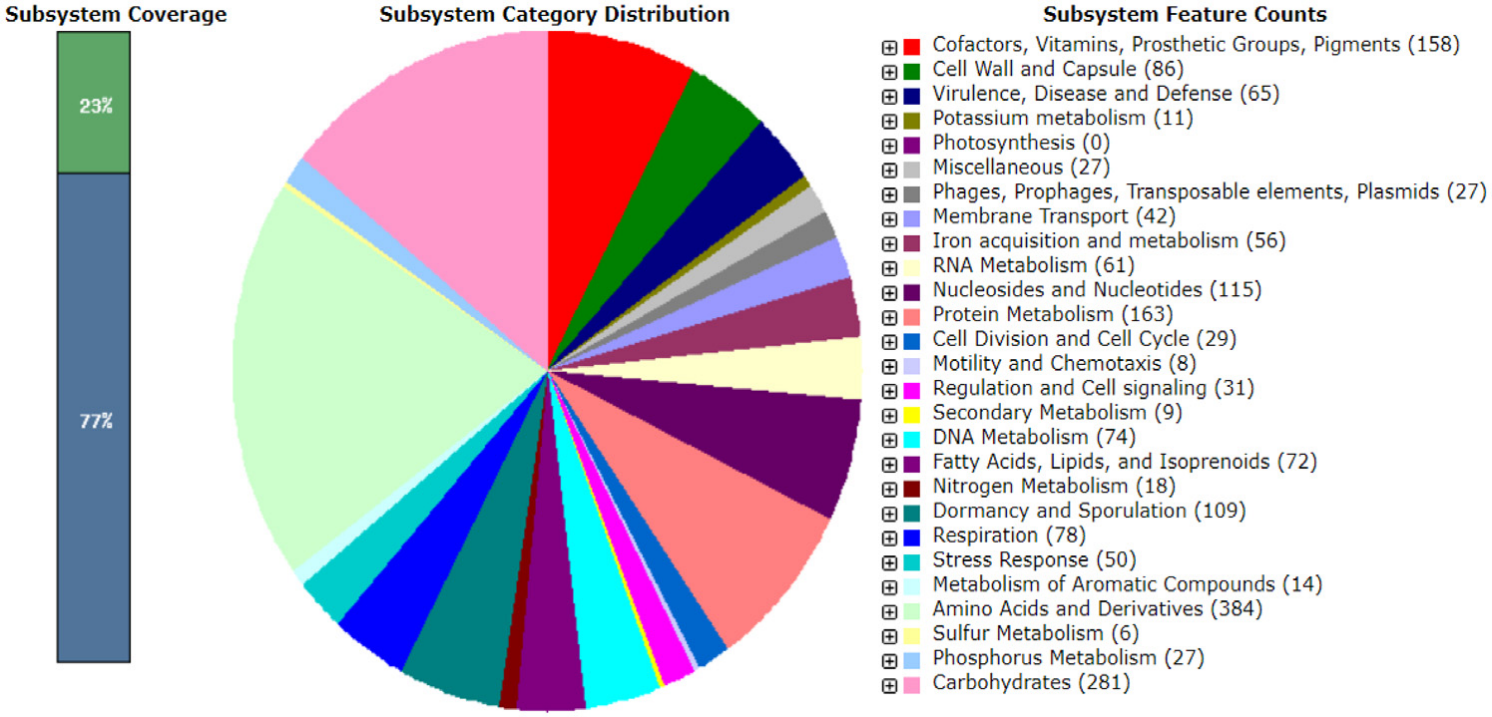
Subsystem statistics information of *Bacillus* sp. CCB-MMP212 using RASTtk annotation. List of Super Classes and its corresponding subsystems features were shown in the legend.

Fig. 2 shows the subsystem statistics information of *Bacillus* sp. CCB-MMP212. The bar chart on the left side of the figure depicts the percentage coverage of subsystems. The pie chart generated by the RAST server and viewed in SEED viewer depicts the distribution of the 27 most common subsystem categories among 2118 subsystem categories. The most abundant subsystem categories were amino acids and derivatives (384), carbohydrates (281), cofactors, vitamins, prosthetic groups, and pigments (158). Interestingly, an *ars* operon consisting of *asrR, I, B*, and *C* was present in the genome (Table 3). Yoshinaga and colleagues reported that trivalent organoarsenicals, such as MAs(III) and Rox(III), are degraded to As(III) by ArsI with C•As lyase activity [6]. Then, As(III) might be released from the cell by an arsenite efflux permease, ArsB. Thus, bacteria with C•As lyase, including CCB-MMP212, might play an important role in arsenic biogeocycle through the degradation of environmental organoarsenicals.

**Table 3:** Arsenic enzyme coding genes found in *Bacillus* sp. CCB-MMP212 genome.

Start	Stop	Strand	Gene	No of Locus		Protein name	Description
348446	348751	+	*ars*R	MCI4251078.1	101	Arsenical resistance operon transcriptional regulator *Ars*R	As(III)-responsive repressor of transcription [1].
348812	349249	+	*ars*I	MCI4251079.1	145	Glyoxalase/bleomycin resistance/dioxygenase family protein	Responsible for MAs(III) demethylation. Cleaves the C·As bond in a wide range of trivalent, organoarsenicals, including the trivalent roxarsone [Rox(III)], into As(III) [3].
349268	350308	+	*ars*B	MCI4251080.1	346	ACR3 family arsenite efflux transporter	Extrude the trivalent arsenic As(III) from the cell [3].
350329	350733	+	*ars*C	MCI4251081.1	134	Arsenate reductase (thioredoxin)	Reduce the arsenate ion (H_2_AsO_4_-) to arsenite ion (AsO2-) [2].

## 2 Experimental Design, Materials and Methods

### 2.1 Sample collection

*Bacillus* sp. CCB-MMP212 was isolated from sediment in Matang Forest Mangrove, Perak, Malaysia. The strain was deposited in the Centre for Chemical Biology-Microbial Biodiversity Library (CCB-MBL) in freeze-dried form and was stored in 40% glycerol stock at −80°C.

### 2.2 DNA Extraction

The DNA extraction was performed according to the method of Sokolov [7] with slight modifications. Bacterial resuspension was spun down and supernatant (ethanol) was removed via decantation. The pellet was resuspended in 500 µL of lysis buffer (50 mM NaCl, 50 mM Tris-HCl pH8, 50 mM EDTA, 2% SDS) and incubated for 30 min at 60°C. A volume of 3 µL RNAse A (10 mg/mL) was added to the lysate and incubated for 10 min at room temperature. A volume of 50 µL (0.1x vol) saturated KCl was added at 4°C for 5 min to remove the salt. The lysate was extracted once with an equal volume of chloroform to remove the remaining proteins. The aqueous layer containing the DNA was mixed with an equal volume of isopropanol and 20 µL of solid-phase reversible immobilization (SPRI) bead to promote the binding of DNA onto the solid carboxylated layer [8]. The mixture was incubated for 10 min at room temperature. Then the mixture was placed on a magnetic rack for 2 min and the supernatant was discarded. The bound magnetic bead was washed twice with 75% ethanol. The bead was resuspended in 100 µL of TE buffer, then incubated at 50°C for 5 min to extract the DNA.

### 2.3 Nanopore and Illumina library preparation and genome sequencing

According to the manufacturer's instructions (Oxford Nanopore, UK), approximately 400 ng of DNA as measured by Qubit was fragmented with the Nanopore rapid barcoding kit. On a Nanopore Flongle flow cell, the sample was sequenced. Guppy v4.4.1 was used to extract the fast5 file (high accuracy mode) [9]. Approximately 100 ng of DNA was fragmented to 350 bp using a Bioruptor, then the NEB Ultra II library preparation kit for Illumina was used according to the manufacturer's instructions (NEB, Ipswich, MA). Each sample was sequenced on a NovaSEQ6000 (Illumina, San Diego, CA), yielding approximately 1 gb of paired-end data (2×150 bp).

### 2.4 Hybrid De novo assembly - Nanopore and Illumina

Raw nanopore reads were quality- and length-filtered to retain reads with scores of 7 or higher that were longer than 2,000 bp. The filtered Nanopore was then used in combination with the Illumina reads for hybrid assembly with Unicycler (default settings) [10]. Contigs shorter than 500 bp were removed, and the filtered assembly was used for further analysis.

## Ethics Statement

NA.

## Credit Author Statement

**Nor Azura Azami:** Methodology, Writing – original draft, Writing – reviewing; **Lau Nyok-Sean:** Methodology and editing; **Go Furusawa:** Supervision, Writing – review & editing.

## Funding Sources

This work was supported by the Short-Term Grant (304/PCCB/6315540) by 10.13039/501100004595Universiti Sains Malaysia awarded to Nor Azura.

## Declaration of Competing Interest

The authors declared that they have no conflicts of interest.

## Data Availability

Bacillus sp. CCB-MMP212, whole genome shotgun sequencing project (Original data) (NCBI).
